# Knowledge and Attitude of Obstetric Care Providers on Partograph and Its Associated Factors in East Gojjam Zone, Northwest Ethiopia

**DOI:** 10.1155/2016/6913165

**Published:** 2016-06-14

**Authors:** Desalegne Amare Zelellw, Teketo Kassaw Tegegne, Girma Alem Getie

**Affiliations:** ^1^Department of Nursing, College of Medicine and Health Sciences, Bahir Dar University, P.O. Box 79, Bahir Dar, Ethiopia; ^2^Department of Public Health, College of Medicine and Health Sciences, Debre Markos University, Debre Markos, Ethiopia; ^3^Department of Nursing, College of Medicine and Health Sciences, Debre Markos University, Debre Markos, Ethiopia

## Abstract

*Introduction*. Universal use of partograph is recommended during labor, to improve maternal and fetal outcome. The aim was to assess knowledge and attitude of obstetric caregivers about partograph and associated factors.* Methods*. Facility based cross-sectional study was conducted on 273 study participants. Study facilities and study units were selected using simple random sampling technique. Midwives, Nurses, Public Health Officers, Medical Doctors, and masters in Emergency Surgery and Obstetric were included in the study. Epi-data and SPSS statistical software were used.* Results*. About 153 (56.04%) and 150 (54.95%) of the obstetric caregivers had good knowledge and favorable attitude about partograph, respectively. Knowledge of partograph was significantly higher among obstetric caregivers that learnt about partograph during their College and who had received partograph on job training (AOR: 2.14, 95% C.I (1.17–3.93)) and (AOR: 2.25, 95% C.I (1.21–4.19)), respectively. Favorable attitude towards partograph was significantly higher among obstetrical caregivers who had training and learnt about partograph during their college (AOR: 3.37, 95% C.I (1.49–5.65)) and (AOR: 2.134, 95% C.I (1.175–3.877)), correspondingly.* Conclusion*. Above half of obstetric caregivers had good knowledge and a favorable attitude on partograph. The provision of on preservice and job training is necessary to improve caregivers' knowledge and attitude.

## 1. Background

The 2013-world health organization (WHO) report showed that over 289,000 mothers died globally, of which developing countries accounted for 99% and sub-Saharan Africa region alone accounted for about 62% [1]. The majority, 70% of death cases, occurred due to obstructed labor and ruptured uterus [2]. The 2011 Ethiopian demographic and health survey (EDHS) indicated that maternal mortality ratio was at 676 maternal deaths per 100,000 live births [3]. In addition, the 2013 WHO report also showed that maternal mortality in Ethiopia was 420 per 100,000 [1].

Prolonged and obstructed labor is one of the five major causes of maternal death which was responsible for 8% of all maternal deaths. An estimated 6.5 million women in the world have obstructed labor each year (2–15 cases/1000 births). It is the most common cause of complications like death and fistula. Approximately, 2–5% of women who experience a prolonged or obstructed labor can develop fistula [3].

WHO report showed that the global maternal deaths resulted from complications of pregnancy and childbirth, especially, in developing countries [4, 5]. From survive childbirth, at least 8 million develop serious morbidities and a further 50 million suffer minor complications [5]. Out of the causes of deaths and complications, obstetric hemorrhage and obstructed labor are common causes and easily preventable by using partograph [6]. Senegal and Mali indicate that the most common reported causes of maternal death were postpartum hemorrhage. Furthermore, obstructed labor was the cause for maternal death [7].

The World Health Organization recommends the universal use of the partograph during labor [8]. It is a cost-effective and affordable tool used to improve monitoring of the progress of labor and maternal and fetal well-being, which later on is used to reduce maternal deaths and complications due to obstructed and prolonged labor conditions [9, 10]. Physicians should set reasonable expectations for labor progress to avoid unnecessary interventions and anxiety [11]. Prolonged latent phase of labor is associated with a higher risk of postpartum hemorrhage, chorioamnionitis, neonatal admission to the intensive care unit, and long hospital stay [12]. Therefore, early detection of abnormal progress and prevention of prolonged labor can significantly reduce maternal mortality and morbidity [2].

Previously, partograph was introduced to illustrate cervical dilation graphically during labor [13] and later it incorporates the action and alert lines [14]. Currently, the Modified WHO Partograph comprises different variables; therefore, the current partograph was designed to monitor not only the progress of labor but also the condition of the mother and fetus during labor. It involves various parameters to assess progress of labor and maternal and fetal conditions during labor. The progress of labor is assessed through cervical dilatation and descent of the head and uterine contractions. On the other hand, fetal condition is monitored by fetal heart rate, color of liquor, and the moulding of the fetal skull. Furthermore, the maternal condition is also assessed through monitoring maternal pulse rate, blood pressure, temperature and urine for volume, protein, and ketone bodies and additional crucial factor in active management of labor is the timing of interventions as and when needed, such as amniotomy, augmentation with oxytocin, caesarean section, or transfer to a higher center [15].

A study done in Southwest Nigeria revealed that obstetric care providers from tertiary level of care were significantly more knowledgeable about the assessments that could be inferred from the partograph. In addition, most of them had received prior training about partograph In addition, most of them had received prior training about partograph and they knew at least one component of the partograph [16]. However, knowledge of the functions of alert line and action line was generally poor as only 16.6% and 24.3% of them had explained the function of alert line and action line, respectively. A higher proportion of study participants from tertiary level of care perceived that partograph utilization can reduce maternal/perinatal morbidity and mortality and it improves the quality of care [12].

There was a differing knowledge level between healthcare providers working in general hospitals and university teaching hospital, the first having a higher level of knowledge [16]. Previous training on partograph was independently associated with the knowledge of obstetrical care providers about the components of the partograph [17]. In North Shoa, Central Ethiopia obstetric caregivers had a good level of knowledge on the partograph and their level of knowledge was significantly associated with working in hospitals and having on-the-job training about partograph [18].

Knowledge of obstetric caregivers' about the partograph at public health facilities of Addis Ababa, Ethiopia, was fair; 96.6% of the study participants correctly mentioned at least one component of it. In this study, 53.3% and 82.6% of the caregivers properly explained the function of alert line and action line, respectively [19]. A study done in Nigeria indicated that at tertiary and general hospitals healthcare providers' knowledge and previous training on partograph were significantly associated with its utilization during labor. Furthermore, lack of detailed knowledge of it, nonavailability of the partograph, and shortage of staff were the militating factors against the use of partograph [16]. Therefore, this study was aimed to assess knowledge and attitude of partograph and its associated factors among obstetric care providers.

## 2. Methods and Materials

### 2.1. Study Setting

The study was conducted from March to July 2015 at Public Health Facilities (health centers and a hospital) in East Gojjam Zone, Amhara Regional State, and Northwest Ethiopia. Debre Markos is the capital of East Gojjam Zone, which is located at 299 Kilometer Northwest of Addis Ababa. In the zone, there are 19 districts/woreda, 101 health centers, and two hospitals.

Health center is a primary healthcare unit (PHCU) with each health center having five satellite health posts. Health centers comprise (1/15,000–25,000 population) and their satellite health posts (1/3,000–5,000 population) that are connected to each other by a referral system. Hospitals can be district/a primary hospital (with population coverage of 60,000–100,000 people), general hospital with population coverage of 1–1.5 million people, and thirdly a specialized hospital that covers population of 3.5–5 million [20].

### 2.2. Study Design

A health facility based cross-sectional study was conducted at public health facilities in East Gojjam Zone.

### 2.3. Study Population

In governmental health facilities of East Gojjam Zone, estimated 1417 health workers (Nurses, Midwives, Public Health Officers, Medical Doctors, and M.S. in Emergency Surgery and Obstetric) were found.

### 2.4. Sampling

The sample size was determined using a single population proportion, assuming a 95% confidence interval and with 5% margin of error and 26.6% of the proportion of proper knowledge on components of partograph taken from a study done in Amhara Region [21]. Based on this *n* = *z* ^2^(*p*(1 − *p*))/*d* ^2^ formula: the sample size was obtained 300. Since the entire population was below 10,000 a reduction formula was used and the sample size was reduced to 248. The final sample size with a 10% nonresponse rate was obtained, 273.

### 2.5. Sampling Techniques

From the total 103 public health facilities in the zone, that is, 101 health centers and two hospitals, 29 health centers and one hospital were selected by simple random sampling technique. The 273 study participants in the sampled health facilities were selected using simple random sampling method with proportionate allocation to size after getting the list of health workers working in labor and delivery care on either a routine or duty program.

### 2.6. Inclusion and Exclusion Criteria

The survey was conducted among all healthcare professionals: that is, all Midwives, Nurses, Public Health Officers, Medical Doctors, and masters in Emergency Surgery and Obstetric who were working in labor and delivery on a regular and/or duty program were included in this study. However, health workers that had a workload during the visit were excluded from this study.

### 2.7. Variables

Sociodemographic characteristics (age, sex, religion, and marital status), working department, health professional qualification, health facility type, training partograph and obstetrics care, and experience are independent variables while knowledge and attitude of the participants on the partographs are the dependent variables.

### 2.8. Data Collection Process

Questionnaires were adapted and modified from related articles to collect data through self-administered questionnaire. The questionnaire mainly focused on sociodemographic characteristics, qualification, types of health facilities, current working department, knowledge and attitude about partograph, and previous obstetric care training. The study participants were instructed how to fill the questionnaire and the data collection took an average of 30–50 minutes. The data were collected by Nurses, Midwives, and Public Health Officer. A total of three Nurses, two midwifes, and one public health officers were involved in the data collection process.

### 2.9. Data Quality Assurance

The questionnaire was pretested on 5% of non-sampled hospitals and health centers. Based on the pretest findings modification was made. Data collectors and supervisors were trained for two days on data collection instruments and sample collection. All selected data collectors had Bachelor of Science degree in their perspective professions to enhance the quality of the data. The investigators and supervisors were made on site supervision during the whole period of data collection. Data were checked for completeness and consistency after each day of data collection by holding a meeting with the data collectors. Data double entry was made for all the questionnaires to enhance data quality.

### 2.10. Measurements

Knowledge of obstetric caregivers about the partograph was measured based on eight knowledge specific questions. Each correct response earned one point, whereas any wrong response attracted no mark and thus the sum score of knowledge was calculated (8 points). The mean score of partograph knowledge (4.44 ± 2.18) was used to decide cutoffs of the rank. Respondents who scored 0–4 points were adjudged as having poor knowledge, whereas those that scored 5–8 points were adjudged as having a good knowledge, respectively (Table 1).

On the other hand, the attitude towards partograph was assessed using attitude scoring method. The attitude score for each of the personnel was obtained by adding up the scores for correct answers given to the ten questions. Based on the overall attitude scores, the respondents' level of attitude of the partograph was rated using a median score of 8.00 ± 3.49 as unfavorable (0–7) and favorable (8–10) (Table 2).

### 2.11. Data Analysis

Data were entered using Epi-data version 3.1, and analysis was performed using SPSS version 20.0 statistical software. Bivariate and multiple models were run to assess any relationship between each independent and the outcome variables. Crude and adjusted odd's ratios were used to identify any association between the dependent and independent variables. Pearson correlation coefficient was used to examine the effect of knowledge on the attitude of participants and vice versa. Level of significance was determined using 95% confidence intervals. Independent variables with *p* value less than or equal to 0.2 at the bivariate level were included in the multiple logistic regression models for the dependent variables to control potential confounding variables.

### 2.12. Ethical Consideration

Ethical approval and clearance were obtained from Debre Markos University, medicine and health science college Ethical Review Committee. A formal letter of permission and support was written to East Gojjam Zone Health Department. Also the zonal health department wrote a permission letter and support to the sampled health facilities. Then it was communicated at each level of the health facilities and each study participant. The purpose of the study was explained to participants, and written informed consent was obtained from each study subject. Confidentiality of information was maintained by omitting any personal identifier from the questionnaires.

## 3. Result

### 3.1. Characteristics of Obstetric Caregivers

In this study, 273 obstetric caregivers participated obtaining a response rate of 100%. More than half, 157 (57.5%), of them were males. The mean age of the study participants was 27.64 (±4.50) years and nearly half, 128 (46.9%), of them were within the age group of 25–29 years. About 198 (72.5%) of the healthcare providers had a diploma educational status.

The majority, 246 (90.1%) of obstetrics caregivers, were working in the health centers and more than one-fifth, 60 (22.0%), of them were Diploma Midwives. One hundred forty-one (51.6%) of the caregivers were working at delivery ward regularly while the rest were working during the night duty and/or on the weekend. Nearly half, 132 (48.4%) of obstetric caregivers, had a maximum of three years of clinical service and only 91 (33.3%) of them had training on obstetric care. However, only 78 (28.6%) of them received in-service or refresher training on partograph directly or indirectly (see Table 3).

### 3.2. Knowledge about Partograph

In this study, more than half, 153 (56.1%), of the obstetric caregivers had good knowledge about partograph. Even though nearly three-fourth, 200 (73.3%), of the obstetric caregivers knew the components of partograph, there was knowledge deficiency in some other aspects. Only the 57 (20.9%) obstetric caregivers exactly knew that plotting on the partograph is started when the cervical dilation is at four centimeters. Furthermore, 133 (48.7%) of them knew the correct function of the alert line on the partograph (see Table 4). To enhance their knowledge about partograph 161 (58.9%) of the care providers want to receive partograph training. About 53.8% of participants used the tool routinely.

**Figure 1:**
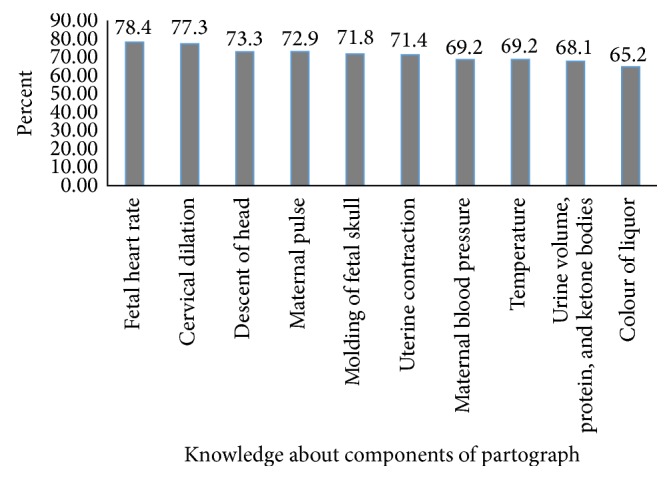
Obstetric caregivers knowledge about components of partograph, East Gojjam Zone, Northwest Ethiopia, 2015 (*n* = 273).

With regard to obstetric caregivers' detail knowledge about the partograph components, 214 (78.4%) of them knew that fetal heart rate (FHR) is one of its components in monitoring the FHR condition. Furthermore, 195 (71.4%) and 186 (68.1%) knew that uterine contraction and urine volume, protein, and ketone bodies are its components in monitoring of the labor progress and maternal conditions, respectively (Figure 1).

### 3.3. Attitude of Obstetric Caregivers towards Partograph

Above half, 150 (55%), of the obstetric care providers in this study had a favorable attitude towards partograph. However, only 111 (40.7%) of the caregivers agreed to use partograph, but 171 (64.1%) of them had agreed that maternal and newborn morbidities and mortalities can be reduced by using partograph. Furthermore, 183 (67%) of the providers agreed that partograph is important, for early detection of labor abnormalities for surgery or caesarean section. A lower proportion, 83 (30.4%), of the caregivers believed that partograph is used only for physicians (medical doctors). Similarly, 107 (39.2%) of them agreed that use of partograph is time consuming and all normal labor does not need to use partograph, 89 (32.6%) (Table 5).

### 3.4. Factors Associated with Partograph Knowledge

According to the multivariable analysis, obstetric care providers within the age of 25–29 years were 2.47 times more probable to have good knowledge about partograph than those below 25 years old (AOR (95% C.I): 2.47 (1.32–4.64)). Obstetric caregivers who were working at antenatal and family planning ward usually were 4.94 and 2.61 times more likely to have good partograph knowledge than those who were routinely working in outpatient department (AOR (95% C.I): 4.94 (1.97–12.40) and 2.61 (1.03–6.61)), respectively. Moreover, caregivers who had learnt about partograph during their college and/or university level of education were 2.14 times more likely to have good knowledge about partograph than their counterparts (AOR (95% C.I): 2.14 (1.17–3.93)). Similarly, obstetric caregivers who had received on-the-job training on partograph were also 2.25 times more likely to have good partograph knowledge than their counterparts (AOR (95% C.I): 2.25 (1.21–4.19)) (see Table 6). However, having obstetric training was a confounding factor on partograph knowledge.

### 3.5. Factors Associated with Attitude towards Partograph

According to the multivariable analysis obstetric care providers, working in a hospital, were 5.04 times more likely to have a favorable attitude towards partograph than those working in health centers (AOR (95% C.I): 5.04 (1.60–15.80)). Similarly, obstetric caregivers who had received on-the-job training on partograph were also 3.37 times more likely to have a favorable attitude towards partograph than their counterparts (AOR (95% C.I): 3.37 (1.76–6.44)). Furthermore, care providers that learnt about partograph college and/or university were 2.13 times more likely to have a favorable attitude towards partograph (AOR (95% C.I): 2.13 (1.17–3.87)) (see Table 7).

Pearson correlation coefficient between knowledge and attitude was (*r* = 0.370). The degree of relationship between the two variables was moderate (see Table 8).

## 4. Discussion

This study found that above half of the caregivers had good knowledge and favorable attitude about partograph and its use. Knowledge of partograph was higher among those within the age of 25–29 years old, regularly working in antenatal and family planning ward, who had partograph training and those who had learnt partograph at their college or university-level education. On the other hand, obstetric caregivers working in the hospital had good knowledge and training on partograph and had a favorable attitude towards partograph utilization. Overall utilization (53.8%) of the partograph was lower than the knowledge and attitude of the care providers.

In this study above half (56.1%) of the obstetric caregivers had good knowledge about partograph. But there is a knowledge gap in specific areas like when to start plotting on the partograph and the functions of the alert line on the partograph. The reported knowledge is closely related to a study done in Amhara Region, Ethiopia [21], but it was lower than another study done in North Shoa, Ethiopia [19]. This good knowledge score might be obtained due to training opportunities or supportive supervisions and the integration of partograph in routine intrapartum care. Moreover, it was true that on-the-job training on partograph had a positive influence on partograph knowledge [18].

In this study, 55% of the obstetric care providers had a favorable attitude towards partograph and its use. This finding was much lower than a study done in Port-Said and Ismailia Cities as more than 90% of the caregivers had positive attitude towards partograph [22]. A study done in Uganda found that the health workers perceived that use of partograph is useful in helping them to detect abnormal labor [23]. However, in Kenya obstetric caregivers had a negative attitude towards using partograph [24]. These differences might be attributed to differences in study area, which might be explained by differing strategies and commitments in implementing the health policy at the various levels throughout the country and different levels of knowledge of the obstetric care providers towards partograph. In addition, the difference in study participants might have a difference in attitude towards partograph.

Knowledge about partograph was significantly higher among obstetric caregivers within the age group of 25–29 years old as compared to those below 25 years. It might be because as the age of individual increases, the probability of acquiring comprehensive knowledge of the partograph would also be increased. This could be related to experience or on-the-job training as refresher training on partograph or obstetric care had a positive relationship in the knowledge and use of the partograph [16]. Moreover, they might be the only one to be consulted by their junior obstetric caregivers and thus might update themselves.

Furthermore, knowledge about partograph was significantly higher among obstetric care providers who had ever received on-the-job training on partograph. In addition, those obstetrics care givers who have had favorable attitude likely to have better knowledge as compare to unfavorable knowledge. This finding was supported by another study done in Southwestern Nigeria, Addis Ababa and North Shoa, Ethiopia [17–19]. This might be due to the fact that obstetric care providers that received on-job training might enhance their better knowledge or understanding of the partograph than others that in turn improves their partograph utilization.

Knowledge of the partograph was significantly associated with participants that learnt partograph on their formal educational curriculum at university or college level, which was supported by a study done in Southwest Nigeria [12]. This University or college-level formal education on partograph might improve their knowledge and skills, later on improving utilization of partograph as supported by another study [16]. Therefore, this brings the need to introduce some form of obstetric/partograph training or continued professional development its value. Those participants who had been working at antenatal care and family planning department were independently associated with knowledge of partograph, though the association was marginally significant in case of participants who worked at family planning department. This might be due to the fact that obstetric caregivers assigned in antenatal care and family planning departments could have a better chance of receiving training on partograph which might improve their knowledge about partograph than others.

Obstetric caregivers' attitude towards partograph was significantly higher among those who were working a hospital. The knowledge and attitude have positive correlation: that is, as the knowledge of the participants increased, the attitude also increased and vice versa. The likely explanation for this might be that obstetric caregivers working in the hospital might have good knowledge as it was corroborated by a study done in Addis Ababa [19]. Furthermore, as it is supported by this study's finding, they might have on-the-job training on partograph which builds a favorable attitude on partograph and the importance of its utilization.

## 5. Conclusion

More than half of the obstetric care providers had good knowledge and favorable attitude about partograph. Partograph knowledge was higher among obstetric caregivers within the age of 25–29 years old, had in-service partograph training, and those who had college and/or university-level education about partograph and who were regularly working at the antenatal care and family planning departments. Those who had on-the-job training on partograph and who had college and/or university-level education about partograph had significant association with favorable attitude. Knowledge had effects to have favorable attitude and vice versa. Therefore, a greater emphasis should be given for preservice and on-job training for obstetric caregivers to improve their knowledge and attitude about partograph.

## Figures and Tables

**Table 1:** Criteria for the partograph knowledge score.

Parameters	No	Yes
Correct definition of partograph	0	1
Mention at least one component of partograph	0	1
The use of partograph	0	1
Know functions of alert line	0	1
Know functions of action line	0	1
Know when to start plotting on partograph	0	1
Importance of partograph	0	1
Know satisfactory labor progress	0	1

**Table 2:** Criteria for the partograph attitude score.

Parameters	Disagree	Agree
Like to use partograph	0	1
Partograph is important to monitor labor	0	1
Partograph should be used in all labor	0	1
Partograph use reduces risks of maternal and/neonatal morbidity and mortality	0	1
Partograph helps for early detection for surgery or cesarean section	0	1
Wish to use partograph routinely	0	1
Not all normal labor needs partograph	1	0
Using partograph is the only responsibility of physicians	1	0
Partograph is not effective to monitor labor progress	1	0
Using partograph is time consuming	1	0

**Table 3:** Characteristics of obstetric caregivers and public health institutions of East Gojjam Zone, Northwest Ethiopia, 2015 (*n* = 273).

Variables	Frequency	Percentage
Sex		
Male	157	57.5
Female	116	42.5
Age in years		
≤24	71	26
25–29	128	46.9
≥30	74	27.1
Health institution		
Health center	246	90.1
Hospital	27	9.9
Qualification level		
Diploma	198	72.5
Degree and above	75	27.5
Profession		
Nurse	155	56.8
Midwife	74	27.1
Public Health Officer	37	13.6
Others^†^	7	2.6
Regular working department		
Delivery ward	141	51.7
Antenatal care	47	17.2
Family planning	36	13.2
OPD (adult & or under-five)	49	17.9
Clinical service years		
≤3 years	132	48.4
4–6 years	87	31.9
≥7 years	54	19.8

^†^Others (medical doctors and M.S. in emergency surgery and obstetrics).

**Table 4:** Knowledge of obstetric caregivers on partograph and its use, East Gojjam Zone, Northwest Ethiopia, 2015 (*n* = 273).

Knowledge variables	Correct response (*n*)	Percentage
Definition of partograph	153	56
Components of partograph	200	73.3
The use of partograph	156	57.1
Functions of alert line	133	48.7
Functions of action line	197	72.2
When to start plotting on partograph	57	20.9
Importance of partograph	163	59.7
Satisfactory labor progress	152	55.7
Overall knowledge		
Good knowledge	153	56.1
Poor knowledge	120	43.9

**Table 5:** Attitude of obstetric caregivers on partograph and its use, East Gojjam Zone, Northwest Ethiopia, 2015 (*n* = 273).

Attitude variables	Response (agreed) (*n*)	Percentage
Like to use partograph	111	40.7
Partograph is important to monitor labor	171	62.6
Partograph should be used in all labor	181	66.3
Partograph use reduces risks of maternal and/neonatal morbidity and mortality	175	64.1
Partograph helps early detection for surgery or CS	183	67.0
Wish to use partograph routinely	174	63.7
Not all normal labors need partograph	89	32.6
Using partograph is the only responsibility of physicians	83	30.4
Partograph is not effective to monitor labor	84	30.8
Using partograph is time consuming	107	39.2
*Overall attitude *		
Favorable attitude	150	55
Unfavorable attitude	123	45

**Table 6:** Factors associated with knowledge of partograph, East Gojjam Zone, Northwest Ethiopia, 2015 (*n* = 273).

Variables	Partograph knowledge		Crude odds ratio (95% C.I)	Adjusted odds ratio (95% C.I)
	Poor	Good		
	*n* (%)	*n* (%)		
Age in years				
≤24	39 (54.93)	32 (45.07)	1.00	1.00
25–29	45 (35.16)	83 (64.84)	**2.25 **(1.24–4.06)^*∗∗*^	**2.47 **(1.32–4.64)^*∗∗*^
≥30	36 (48.65)	38 (51.35)	1.29 (0.67–2.47)	1.76 (0.86–3.59)
Regular working department				
Outpatient department	29 (59.18)	20 (40.82)	1.00	1.00
Delivery ward	63 (44.68)	78 (55.32)	1.80 (0.93–3.47)	1.74 (0.87–3.52)
Antenatal care	13 (27.66)	34 (72.34)	**3.79 **(1.61–8.93)^*∗∗*^	**4.94 **(1.97–12.40)^*∗∗*^
Family planning	15 (41.67)	21 (58.33)	2.03 (0.85–4.86)	**2.61 **(1.03–6.61)^*∗*^
Obstetric training				
No	88 (48.35)	94 (51.65)	1.00	1.00
Yes	32 (35.16)	59 (64.84)	**1.73 **(1.03–2.90)^*∗*^	0.98 (0.45–2.15)
Partograph training				
No	98 (50.26)	97 (49.74)	1.00	1.00
Yes	22 (28.21)	56 (71.79)	**2.57 **(1.46–4.54)^*∗∗*^	**2.25 **(1.21–4.19)^**∗**^
Learnt partograph				
No	48 (59.26)	33 (40.74)	1.00	1.00
Yes	72 (37.50)	120 (62.50)	**2.42 **(1.43–4.12)^*∗∗*^	**2.14 **(1.17–3.93)^**∗**^
Profession				
Nurse	70 (45.2)	85 (54.8)	1.00	
Midwifes	32 (43.2)	42 (56.8)	1.08 (0.61–1.88)	
Public Health Officer	15 (40.5)	22 (59.5)	1.20 (0.58–2.50)	
Others	3 (42.9)	4 (57.1)	1.09 (0.23–5.07)	
Clinical service years				
≤3 years	55 (41.7%)	77 (58.3%)	1	
4–6 years	35 (40.2%)	52 (59.8%)	1.06 (0.61–1.84)	
≥7 years	30 (55.6%)	24 (44.4%)	0.57 (0.30–1.08)	

Significant at ^*∗*^ *p* value < 0.05 and ^*∗∗*^ *p* value < 0.01. Others: medical doctor and masters of emergency and emergency obstetrics.

**Table 7:** Factors associated with attitude towards partograph, East Gojjam Zone, Northwest Ethiopia, 2015 (*n* = 273).

Variables	Attitude towards partograph		Crude odds ratio (95% C.I)	Adjusted odds ratio (95% C.I)
	Unfavorable	Favorable		
	*n* (%)	*n* (%)		
Health facilities				
Health center	119 (48.37)	127 (51.63)	1.00	1.00
Hospital	4 (14.81)	23 (85.19)	**5.39 **(1.81–16.04)^*∗∗*^	**5.04 **(1.60–15.80)^*∗∗*^
Regular working department				
Outpatient department	31 (63.27)	18 (36.73)	1.00	1.00
Delivery ward	53 (37.59)	88 (62.41)	**2.86 **(1.46–5.61)^*∗∗*^	**2.26 **(1.09–4.65)^*∗∗*^
Antenatal care	23 (48.94)	24 (51.06)	1.80 (0.80–4.06)	1.97 (0.82–4.72)
Family planning	16 (44.44)	20 (55.56)	2.15 (0.90–5.18)	2.53 (0.99–6.43)
Obstetric training				
No	96 (52.75)	86 (47.25)	1.00	1.00
Yes	27 (29.67)	64 (70.33)	**2.65 **(1.55–4.52)^*∗∗∗*^	1.32 (0.59–2.94)
Learnt partograph				
No	53 (65.43)	28 (34.57)	1.00	1.00
Yes	70 (35.46)	122 (63.54)	**3.30 **(1.92–5.68)^*∗∗∗*^	**2.13 **(1.17–3.87)^*∗∗*^
Partograph training				
No	105 (53.85)	90 (46.15)	1.00	1.00
Yes	18 (23.08)	60 (76.92)	**3.89 **(2.14–7.07)^*∗∗∗*^	**3.37 **(1.76–6.44)^*∗∗∗*^
Profession				
Nurse	80 (51.6)	75 (48.4)	1.00	1.00
Midwifes	27 (36.5)	47 (63.5)	**1.85 **(1.05,3.27)^**∗**^	0.58 (0.27–1.27)
Public health officer	15 (40.5)	22 (59.5)	1.56 (0.75–3.24)	1.62 (0.74–3.53)
Others	1 (14.3)	6 (85.7)	6.40 (0.75–54.41)	0.62 (0.04–8.11)
Clinical service years				
≤3 years	54 (40.9%)	78 (59.1%)	1	
4–6 years	44 (50.6%)	43 (49.4%)	0.68 (0.39–1.17)	
≥7 years	25 (46.3%)	29 (53.7%)	0.80 (0.42–1.52)	

Significant at ^*∗*^ *p* value < 0.05, ^*∗∗*^ *p* value < 0.01, and ^*∗∗∗*^ *p* value < 0.001.

**Table 8:** Degree of relationship between the knowledge and attitude of the obstetrics care providers.

	Pearson correlation		
	Knowledge	Attitude	*p* value
Knowledge	1	0.370^*∗∗*^	0.000
Attitude	0.370^*∗∗*^	1	0.000

^*∗∗*^Correlation is significant at the 0.01 level (2-tailed).
